# Water carrying in hills of Nepal–associations with women’s musculoskeletal disorders, uterine prolapse, and spontaneous abortions

**DOI:** 10.1371/journal.pone.0269926

**Published:** 2022-06-23

**Authors:** Regula Meierhofer, Vica Marie Jelena Tomberge, Jennifer Inauen, Akina Shrestha

**Affiliations:** 1 Department of Sanitation, Swiss Federal Institute of Aquatic Science and Technology (Eawag), Water and Solid Waste for Development (Sandec), Dübendorf, Switzerland; 2 Department of Health Psychology & Behavioral Medicine, Institute of Psychology, University of Bern, Bern, Switzerland; 3 Kathmandu University, School of Medical Sciences, Kathmandu, Nepal; International Medical University, MALAYSIA

## Abstract

More than a third of women in Nepal have to carry water from source to home to satisfy their families’ daily needs. A cross-sectional study was carried out in a hilly area in Nepal to assess water-carrying practices and their association with women’s health. Quantitative interviews were conducted with 1001 women of reproductive age and were complemented with health surveys carried out by health professionals and structured observations of water carrying. Multivariate mixed logistic regression models were used to assess the associations between water-carrying-related risk factors and health issues for women. Around 46% of women faced considerably increased to excessive physical stress due to water carrying during the dry season. Women suffered from a disproportionately high prevalence of back pain (61%), with about 18% of this pain being horrible to excruciating; pain in the knees (34%); uterine prolapse (11.3%); and at least one spontaneous abortion (9%). The risk category of water carrying was significantly associated with uterine prolapse (OR = 1.44, 95%CI = 1.12–1.85, *p* = 0.031) and pain in the hips (OR = 1.69, 95%CI = 1.27–2.26, *p*<0.001). Receiving help with water carrying during pregnancy and during the first three months after delivery was associated with reduced odds ratios for uterine prolapse (OR = 0.10, 95% CI = 0.01–0.87, *p* = 0.037), and strong back pain (OR = 0.32, 95% CI = 0.12–0.87, *p* = 0.026). Improvements to water supply infrastructure and the promotion of social support for carrying water during pregnancy and after delivery are recommended to reduce water-carrying-related health risks.

## Introduction

In July 2010, the UN General Assembly recognized the right to sufficient and safe drinking water as a human right that is essential for the full enjoyment of life [1]. Access to sufficient water is linked to having the water source in the house or on the plot which leads to improved water quality and increased water quantity, resulting in improved hygiene practices and various health benefits [2,3]. Yet although 71% of the global population used a safely managed drinking water source in 2015, 2.1 billion people globally still lack access to a safe water supply at home [4]. In Nepal, 95% of the population had access to basic water supply services, while 69% of the households in rural areas had water available on their premises in 2017 compared to 38% in 2000 [5,6].

Since the responsibility for ensuring a sufficient supply of water to meet daily household demand lies mostly on women’s shoulders, access to water that is a distance away results in a considerable burden of time and energy for women who carry the water home [7,8]. This work is directly linked to women’s health because it can lead to musculoskeletal pain, fatigue, and emotional stress [9,10]. Geere et al. conducted a systematic review of 42 studies on the association between water carrying and health and found moderate quantitative and strong qualitative evidence that water carrying is associated with musculoskeletal pain, fatigue, perinatal health problems, and violence against vulnerable people. Musculoskeletal disorders due to water carrying were the most likely of these conditions to be reported [9].

Systematic evidence on the association between water carrying and perinatal health problems is limited. Issues identified relate to the link between the need to carry water and the use of prenatal health services [11]. A qualitative study documented women’s involvement in carrying water from distant sources during pregnancy due to social pressure from their mothers-in-law [12]. The necessity to provide water for daily consumption and difficult water supply conditions in low-income areas put women under pressure to carry heavy loads even during vulnerable periods of their life such as during pregnancy and after delivery [13,14]. Carrying and lifting loads during these times pose an increased risk of health problems, such as musculoskeletal disorders due to reduced load-bearing capacity associated with joint laxity [15], spontaneous abortions, preterm delivery, and pelvic organ prolapse [16–18].

In Nepal, pelvic organ prolapse is a significant public health problem [19]; the UNFPA has estimated the national prevalence at 6–10% [20–22]. This compares to prevalence estimates of 3–6% for pelvic organ prolapse in the USA and Europe when the prolapse is defined by symptoms [23]. Extensive physical work during pregnancy and after delivery have been suggested as risk factors for uterine prolapse in Nepal [24–26] and other low-income countries [27–29]; these are in addition to vaginal child birth, advancing age, and increasing body mass index [30]. Similarly, frequent lifting of heavy loads during pregnancy have been identified as potential risk factors for spontaneous abortions [16,17,31] but evidence on the association between carrying water specifically and uterine prolapse or spontaneous abortions has not been assessed yet.

**Fig 1:**
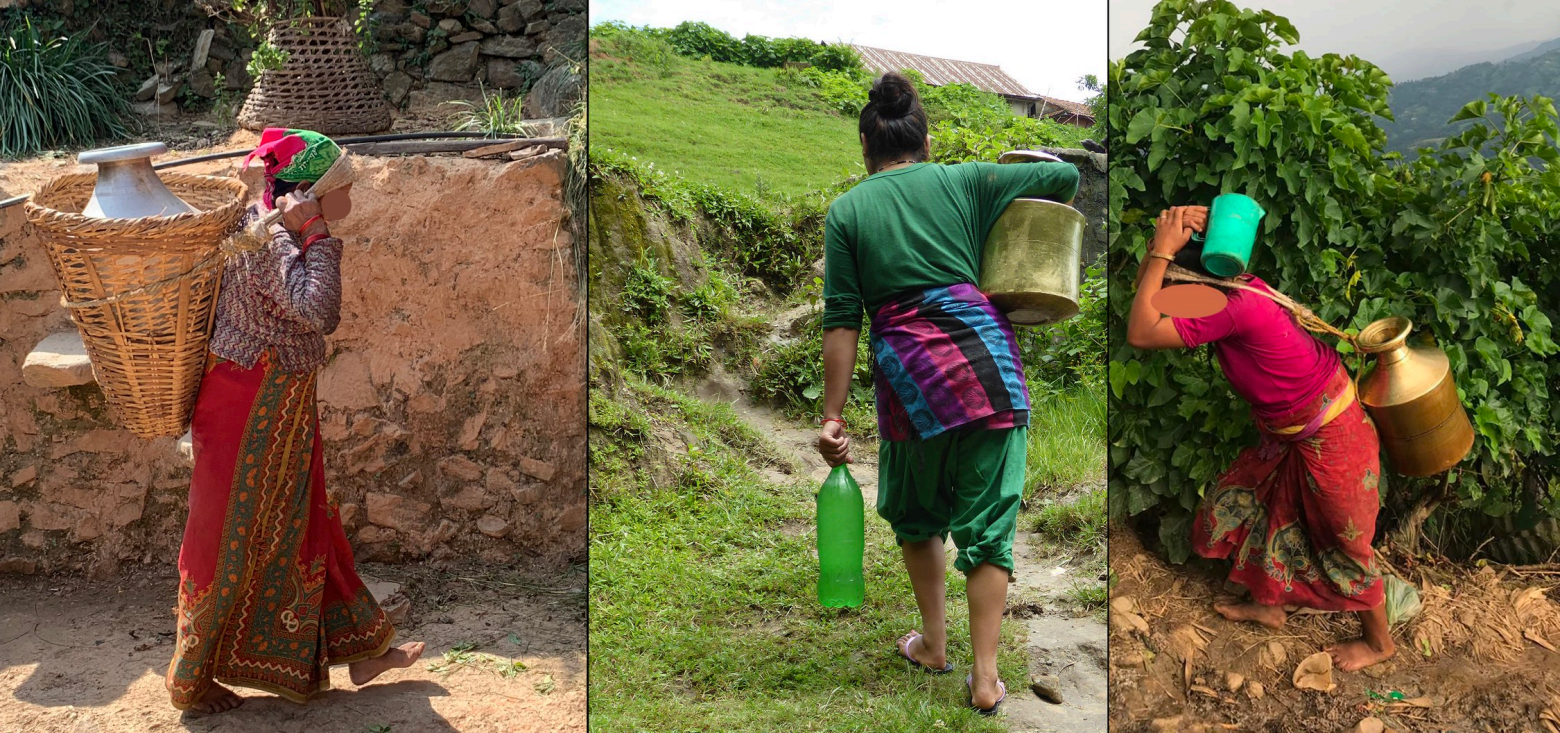
Water carrying with basket (doko) and straps (namlo) and on the waist.

In spite of its relevance, research on the multiple impacts of water carrying on women’s health is not extensive [7]. Therefore, our study aimed at complementing existing evidence with more in-depth quantitative data on water-carrying conditions, practices, and responsibilities, including a special focus on practices during vulnerable periods, such as pregnancy and after delivery. We further focused on assessing the association of water carrying related risk factors with musculo-skeletal disorders, uterine prolapse and spontaneous abortions among water-carrying women in the reproductive age in the hills of Nepal. Sorensen at al. noted the need to investigate the association between the terrain and water carrying [7]. Due to their topography, which might increase some water carrying related risk factors, the hills in Nepal offer ideal conditions to assess the association between up- or downhill water carrying and health. In Nepal, women commonly carry water containers either on the waist or with straps (namlo) tied around the containers directly or around baskets (doko) with containers in them and around the forehead (Fig 1). We therefore, hypothesized that the back, neck, and hips may be particularly affected by carrying water loads.

## Methods

### Study design and procedures

Data for this cross-sectional study were collected from September to November 2019 in the Kavre and Sindhupalanchowk Districts of Nepal. The five study sites, Salambhu, Bolde, Baluwa, Bahunipati, and Kattike Deurali, are located in the mid-hills and represent typical conditions for mountainous areas at an elevation range of 550 to 2000 meters above sea level. They were selected purposively to represent a variety of water supply conditions, including the availability of water connections in the households, on-plot, in close vicinity, and sites with more distant water sources, therewith exposing women to different potential risk factors for the targeted health outcomes. The selected sites included outreach centers of Dhulikhel hospital. In each of the five communities, 200 women of reproductive age were randomly selected using the random route method [32].

Survey instruments included a quantitative questionnaire, a structured observation and a health examination. The questionnaire, the observation form and the health examination form are presented in file S1 Questionnaire, observation and health examination form in the supplementary materials. The survey instruments were translated from English to Nepali and back-translated, and they were pretested in the Fulbari community of Namobuddha municipality in settings similar to those of the study. About 20 women were selected randomly and interviewed. Changes were made to psychological questions in the questionnaire to enhance their understanding. Participants from the pretest were not included in the main study.

Trained interviewers conducted quantitative face-to-face interviews with a total of 1001 women of reproductive age. Sample size and statistical power were calculated using G*Power 3.1. A sample size of 200 interviewees was required at each of the five sites to detect a small to medium effect in Cohen’s f^2^ of 0.04 at alpha of 0.05 and a statistical power of 95% with multivariate regression and 32 predictor variables adjusting for the random effect at the study site [33,34]. The selection criteria were age between 16 to 50 years, residence in the study areas and having provided written informed consent to participate in the study. For the 13 women who were 16 to 17 years of age, additional oral consent was requested from their husband or mother-in-law. The questionnaire provided self-reported information on health outcomes such as musculoskeletal disorders, uterine prolapse, and spontaneous abortion, and on potential confounding and explanatory variables as explained in the section “Variables and Measurements” below. The questions were coded in the open data kit software (ODK, University of Washington, Seattle WA, USA) on tablets (Samsung Galaxy note 10.1 N8010, Seoul, Korea). The questionnaire contained closed-ended questions with numerical, binary (presence, absence), and categorical answer categories.

After the interview, the interviewers accompanied the women during water collection. During the structured observation, the interviewers used a GPS (Garmin CSX 60 and Garmin dtrex h) to measure the distance and altitude difference between the water source and the household. Using a structured observation form, the interviewers rated the physiological behavior of lifting and carrying water containers by assessing whether women kept their backs straight during lifting and lowering the loads, whether they bent their knees while lifting and whether they twisted their backs while lifting. The weight carried was measured using a scale. Information gathered during the observation of the water-carrying practice was recorded on the structured observation form coded on ODK. Pregnant women, women who had delivered less than six months prior to the survey and women with health complaints were requested to be very careful and not to demonstrate anything beyond their daily currently practiced burden and only to demonstrate their practice if they felt well at the moment of the visit.

In addition, health professionals measured the height and weight of all women included in the study and asked them about symptoms and treatment measures for uterine prolapse and about the prevalence of abortions. The findings of the health examinations were recorded in a structured data collection sheet coded on ODK.

The study was presented to village officials to obtain their prior approval. The study participants were informed about the purpose and the procedure of the study and provided written informed consent before the interviews were conducted. Study participants not able to sign indicated consent with their thumbprint The study protocol was reviewed and approved by the Ethical Review Board of the Nepal Health Research Council (Reg No. 517/2019) and the Ethics Commission of the University of Bern (No 2019-10-00003).

### Variables and measurements

#### Outcome variables

Data on self-reported musculo-skeletal disorders were collected during the quantitative interview which contained questions on the presence or absence of pain at the back, neck, head, hips, knees, ankles, feet, muscles of the arms, shoulders, elbows, joints of hands and muscles of the legs.

For back pain and neck pain, verbal rating scales on intensity and frequency of pain were developed in addition to binary answer categories [35] because previous studies had indicated an association between water carrying and these health disorders but very few studies previously had reported findings on the association between water carrying and pain based on an intensity scale [9]. For those women who indicated the presence of back pain or neck pain, the intensity of pain was categorized on a 5-point scale ranging from mild to excruciating. For regression analysis the intensity scale of back pain was regrouped into a binary variable consisting of 0 = no pain, mild pain or discomforting pain versus 1 = distressing, horrible or excruciating pain. The frequency of pain was categorized into a 3-point scale: occasionally, frequently, and constant. Self-reported pain intensity scales have been shown to be valid and reliable to evaluate pain severity caused by a range of medical conditions [35,36]. Strong back pain, pain in the hips, pain in the neck and pain in the knees were selected as outcome variables for the regression models due to the water carrying technique, which causes a higher stress on this part of the body, or due to a higher prevalence of this health outcome in the study population.

Information on the prevalence of uterine prolapse and the degree of prolapse was collected from the participants through questions being asked during the health examination by the health professionals (nurses) during the interview at the interviewees’ homes. The criteria used for the classification of the degree of prolapse applied by the nurses were for stage I: having the sensation of something slipping down a little in the vagina, but feeling nothing in the vaginal opening; stage II: something has slipped down to the level of the vaginal opening; stage III: the uterus has dropped down so much that up to 1 cm of it is bulging out of the vaginal opening; stage IV: more than 1cm of the uterus is bulging out of the vaginal opening, having difficulty to walk and seeking health services due to this. Women having a problem with uterine prolapse were advised to visit the local health centers for further examinations. The interviewees were also offered treatment free of cost for third and fourth degree prolapse in Dhulikhel Hospital.

Data on the type and prevalence of abortions was gathered during the interview by the health professionals. Spontaneous abortion was defined as a recognized spontaneous loss of the embryo or fetus during the pregnancy. Detailed measurements of outcome variables are provided in S2 Table measurements of variables in the supplementary materials.

#### Explanatory variables

Variables included in the regression models controlled for confounding by:

- demographic factors (age, education of interviewee and husband, wealth). The calculation of the wealth index is explained in the section on statistical analysis.
- the weight and frequency of carrying loads other than water,
  And the model included known and suspected risk factors related to:
- the practice of carrying water (age of starting to carry loads, risk category of water carrying, difference in altitude between the water source and the home, using a basket and straps to carry the water container, help received during the daily water transport)
- reproductive health (age, age at birth of the first child, parity, the body mass index (BMI), if the women carried the same amount of water during pregnancy or carried the same amount of water during the first three months after delivery, help received to carry loads during pregnancy and the first three months after delivery from different family members).

The selected water carrying related risk factors were chosen on the basis of previous evidence of their association with musculo-skeletal disorders, uterine prolapse and spontaneous abortions [9,16,17,24,27,37,38], including guidelines for working safety for manual lifting [39–42].Safety guidelines for manual lifting highlighted the following lifting related stress factors: weight, positioning of the weight, horizontal distance to weight, frequency of lifting, height of lifting, twist during lifting, lateral bending and stability while lifting [41].

The water-carrying-related risk factors included in our study provided self-reported information on the weight carried, walking time to the water source, frequency of collection, and observed information on body posture during the lifting of the loads. The weight of water carried was assessed by asking participants about the number of different volumes of water containers carried. The total weight then was computed considering the maximum and minimum weight carried during each season. Although the volumes of water carried were also recorded during the observation, the self-reported variable was used during the analysis to be able to distinguish between the burden of carrying water during the rainy and dry season. Variables used to calculate body posture were recorded during the observation in accordance with [39,41,42] and included whether the back was kept straight or not during lifting, and whether the body was twisted or not while lifting the load. The water carrying related risk factors were condensed into risk categories for water carrying [43] following the procedure presented in the chapter below.

The difference in altitude covered during carrying was assessed during the observation by measuring the altitude at the home and at the water source via GPS and calculating the difference. Also, the method of carrying the container (using straps and a basket (doko) to carry the container or carrying the container on the waist) was noted during the observation. The difference in altitude and method of water carrying were included in the regression analysis to assess their potential association with women’s health although no previous evidence was identified in literature on associations between the type of terrain walked or the use of straps for container carrying and the selected health outcomes.

The transport and lifting of weights during pregnancy and during the first six months after delivery previously had been identified as risk factors for uterine prolapse and spontaneous abortions [16,17,23,24,27,31] and, therefore, were included in the regression analysis. Women were asked if they carry the same amount of water during this period and from whom they had received help with lifting and carrying.

The body mass index (BMI) was included in the study as a potential confounding factor as malnourishment or overweight could modify the association between water carrying and the assessed health outcomes [39,41]. The BMI was calculated in accordance with WHO’s definitions as weight in kilograms divided by height in meters squared (kg/m^2^) and then coded into underweight (<18.5), normal (18.5–24.9) and overweight (≥25.0) [44,45].

Detailed measurements of explanatory variables are provided in S2 Table measurements of variables in the supplementary materials and in S1 Questionnaire, observation and health examination form in supplementary materials

### Risk categories for water carrying

Risk categories relating to the lifting and carrying of weight were defined in accordance with the approach described by SUVA, the Swiss National Accident Insurance. Two risk categories were calculated: the risk of carrying water during the rainy season and the risk of water carrying during the dry season. The following formula was used [43]:

Risk score (rainy season or dry season) = (weight valuation + body posture valuation + execution valuation) × time valuation

Weight valuation = Categorization of maximum self-reported water weight carrying during the rainy season or dry season in accordance with [43].

Body posture valuation = Categorization of keeping the back straight or not, bending knees or not, twisting the back or not while lifting the load in accordance with [43].

Execution valuation = Categorization of ergonomic conditions while lifting and carrying the load = 1 in accordance with [43].

Time valuation = Categorization of number of self-reported trips to water source per day × self-reported minutes to reach water source during the rainy season or dry season in accordance with [43].

Detailed measurements of the variables used to calculate the risk score are provided in S2 Table Measurements of Variables in the supplementary materials. For the execution valuation a score of 1 was used for all study participants as the standing stability was reduced due to the carrying being executed on uneven, soft ground.

The risk score was coded into four categories: Category I: light stress—harm to health not very likely; Category II: increased stress—physical overstraining for less robust individuals possible, measures for improvement recommended; Category III: considerably increased stress—physical overstraining also possible for robust individuals, measures for improvement necessary; Category IV: excessive stress—physical overstraining likely even for trained professionals, measures for improvement necessary (43). During the statistical analysis, the risk category was treated as continuous variable.

### Statistical analysis

The statistical analysis was performed using IBM SPSS Statistics Version 26. Descriptive statistics were calculated to assess the prevalence of health outcomes and frequencies of risk factors. Numerical variables were described by means and standard deviations, and categorical variables were described by absolute and relative frequencies.

We assessed the association of six health-related outcome variables and potential risk factors using multivariate generalized linear mixed models (GLMM) with a binomial probability distribution, logit link and random intercepts to account for clustering at the five village sites. Separate models were run for each outcome variable, i.e. uterine prolapse, spontaneous abortion, strong back pain, neck pain, hip pain, and pain in the knees. The potential risk factors included were: water-carrying-related risk factors, carrying of other loads, water carrying during pregnancy and after delivery, social support for daily water carrying, and social support for water carrying during pregnancy and after delivery. In addition to strong back pain, neck pain, and hip pain, knee pain was selected as an outcome variable due to a high prevalence of knee pain in the study population. The models were adjusted for potential confounding by age, education, body mass index (BMI), wealth index, parity, and age at birth of first child. We present the results of the multivariate GLMM model including the water-carrying-related risk category during the rainy season as predictor, because univariate analysis revealed that more health outcomes were significantly associated with the water-carrying-related risk category during the rainy season than during the dry season. Values of the Variance Inflating Factor (VIF) of all factors included in the model were between 1.06–2.385 and Tolerance was between 0.419–0.943, confirming the validity of model assumptions for multi-collinearity. Chi-square and Spearman’s rho were used to assess correlations between predictors included in the model. The highest correlations were found between the amount of water carried during pregnancy and the amount of water carried during the first three months after delivery (*r*_*s*_ = 0.688, *p*<0.001) and between help received from husbands for carrying loads during pregnancy and after delivery and no help received during this period (*r*_*s*_ = -0.52, *p*<0.001) indicating no problem with collinearity. Therefore, all factors were retained in the model. Odds ratios in the final models were considered as statistically significant if *p*-values were < 0.05. Details of measurements of the variables included in the models are provided in S2 Table Measurements of Variables in the supplementary materials. Due to missing values in several explanatory variables, a sample size of n = 636 was available for the regression models. This enabled the detection of a small to medium effect in Cohen’s f^2^ of 0.06 at alpha of 0.05 and a statistical power of 95%. A student’s *t*-test was used to assess the difference of water access between the dry and rainy season (walking time in minutes to the water source).

Principle component analysis with orthogonal rotation (varimax) was used to calculate a wealth index for interviewees [46,47]. The Kaiser Meyer Olkin (KMO) value was 0.71, indicating a good sampling adequacy. The following items with KMO values above 0.5 were included in the analysis: possession of a radio, TV, mobile phone, bicycle, motor bike, car, and watch; the type of fuel mainly used for cooking; the number of rooms in the house; and the family’s average monthly expenditure. Four components had eigenvalues over Kaiser’s criterion of 1 and together explained 47% of the variance. The wealth index was calculated using the first four components in accordance with previously described procedures [46,47].

## Results

### General demographics

Frequency statistics on general demographics of the women interviewed revealed that 31.6% of the husbands earned their income in agriculture, 30% were employed, 16.7% were engaged as daily laborers, and 12.7% ran small businesses. The majority of women (82%) had received an informal (26.2%), primary (19.6%), secondary (22.4%), or higher (13.9%) education. Nevertheless, poverty levels were high, with 93.0% of the households in the lowest or second lowest quintile of the wealth index. Most women (78.6%) were living together with their husband: They had a mean age of marriage of 18.5 years (SD = 3.6). The mean age at birth of the first child was 19.9 years (SD = 3.7). The mean number of pregnancies was 2.6 (SD = 1.7) and the mean child mortality was 0.16 (SD = 0.47) child deaths, whereby 11.1% of women had lost one child and 2.1% had lost more than one child. More details on demographic information are presented in S3 Table Demographics in the supplementary materials.

### Women’s health condition

Study findings on the prevalence of the health problems found among women in the reproductive age in the study area are presented in Table 1. About 18% of the women suffered from horrible to excruciating back pain. More than 12% of the women had experienced one or more spontaneous abortions, and 11.3% reported symptoms of uterine prolapse. The prevalence of both neck pain (2.9%) and pain in the hips (6.4%) was lower than expected in view of the practice of carrying the water containers on the waist or with straps tied around the head. The prevalence of pain in the knees among women in the reproductive age was 33.7%.

**Table 1:** Prevalence of women’s self-reported health problems.

	Age categories	16–29 years	30–39 years	40–50 years	Total
	*n*	*350*	*323*	*328*	*1001*
Back pain	%	54.7	58.4	70.3	61.0
Back pain distressing, horrible or excruciating		11.7	20.7	22.3	18.1
Back pain frequent to constant	%	13.8	22.0	32.0	21.7
Neck pain	%	0.6	3.4	4.9	2.9
Neck pain distressing, horrible or excruciating	%	0.3	1.9	2.7	1.6
Neck pain frequent to constant	%	0.7	5.5	7.6	3.7
Pain hips	%	6.6	5.3	7.3	6.4
Pain head	%	13.7	16.4	13.7	14.6
Pain knees	%	24.3	32.5	44.8	33.7
Pain muscles arms	%	7.4	15.2	20.4	14.2
Pain shoulders	%	4.6	6.2	10.1	6.9
Pain elbows	%	2.0	5.0	7.0	4.6
Pain joints of hands	%	5.7	6.5	16.3	9.6
Pain fingers	%	0.0	0.6	0.6	0.4
Pain muscles legs	%	5.7	12.1	15.5	11.0
Pain ankles	%	8.0	9.9	14.3	10.7
Pain feet	%	1.4	4.0	2.7	2.7
Had one spontaneous abortion	%	7.1	9.1	11.7	9.5
Had more than one spontaneous abortion	%	1.4	3.6	4.6	3.4
Had one or more abortions	%	8.6	12.8	16.3	12.8
Uterus Prolapse (HS)	%	6.0	9.9	18.3	11.3
Degree of Uterus Prolapse					
Uterus Prolapse first degree	%	4.9	7.5	14.1	8.7
Uterus Prolapse second degree	%	0.6	1.6	2.1	1.4
Uterus Prolapse third degree	%	0.6	0.6	1.2	0.8
Uterus Prolapse fourth degree	%	0.0	0.3	0.9	0.4

### Water access and daily water-carrying practices

In the study area, 88.8% of the interviewees were mainly responsible for carrying water to satisfy their household needs. Help with daily water transport was mainly provided by husbands (44.3%), mothers-in-law (18.2%), daughters (24.5%), and sons (19.5%).

Water access was significantly better during the rainy season than during the dry season, with 68.4% of the women reaching their water source in less than 2 minutes during the rainy season compared to 45.5% during the dry season (*t*(1000) = -13.4, *p* < 0.001). About 10% of the women had to collect water from a source further away than 20 minutes in the dry season and 2.3% in the rainy season. During the rainy season, 48.5% of the women had access to the water source in the house or on the compound, compared to 32.1% during the dry season. Having more difficult water access to water in the dry season resulted in 32.3% of the women stating that they had to carry full water containers uphill from the source compared to 15.9% during rainy season. About 32.7% of the interviewees stated that they conducted more than two trips to carry water per day in the rainy season and 36.1% of the interviewees conducted more than two trips in the dry season. The mean minimum weight carried during the rainy season was 17.9 kg (SD = 8 kg), the mean maximum weight carried during the dry season was 24.0 kg (SD = 10.3 kg). In all, 11.0% of the women were in the highest risk category for water carrying during the rainy season and 23.1% during the dry season (Table 2).

**Table 2:** Access to water source and water-carrying practices.

	N	%	Mean (SD)
**Access to the drinking water source**			
Use a different drinking water source during dry season	1001	39.0	
Minutes to main source in rainy season	1001		3.5 (6.1)
0 minutes		17.4	
1–2 minutes		51.0	
3–5 minutes		18.8	
6–10 minutes		6.7	
11–15 minutes		2.9	
16–20 minutes		0.9	
> 20 minutes		2.3	
Minutes to main source in dry season	1001		17.7 (17.3)
0 minutes		10.9	
1–2 minutes		34.6	
3–5 minutes		19.2	
6–10 minutes		12.2	
11–15 minutes		7.9	
16–20 minutes		5.1	
> 20 minutes		10.2	
Δ Altitude between water source and home (in meters) measured during survey			
< -20m		9.4	
-6 to -20m		9.8	
-2 to -5m		6.6	
-1 to 1m		55.0	
2 to 5m		6.7	
6 to 20m		8.8	
> 20m		3.7	
**Water-carrying practices**			
1 trip per day to main source in rainy season	341	34.1	
2 trips per day to main source in rainy season	331	33.1	
More than 2 trips per day to main source in rainy season	327	32.7	
Nr of trips per day to main source in dry season	306	30.6	
2 trips per day to main source in rainy season	332	33.2	
More than 2 trips per day to main source in rainy season	361	36.1	
Minimum weight carried in rainy season in kg	987		17.9 (8.0)
Minimum weight carried in dry season in kg	987		18.7 (8.1)
Maximum weight carried in rainy season in kg	987		23.3 (10.3)
Maximum weight carried in dry season in kg	987		24.0 (10.3)
Water lifting & carrying technique			
Keeping the back straight while lifting	896	28.9	
Bending knees while lifting	896	39.3	
Twisting while lifting	896	37.5	
Using straps and a basket to carry the water container	896	10.3	
Risk category* maximum water lifting & carrying during rainy season	887		
Category 1		47.6	
Category 2		25.6	
Category 3		15.8	
Category 4		11.0	
Risk category* maximum water lifting & carrying during dry season	887		
Category 1		31.0	
Category 2		22.5	
Category 3		23.3	
Category 4		23.1	

*Category 1: light stress, harm to health not very likely.

Category 2: Increased stress, physical overstraining for less robust individuals possible. Measures for improvement recommended.

Category 3: Considerably increased stress, physical overstraining also for robust individuals possible. Measures for improvement necessary.

Category 4: Excessive stress, physical overstraining likely even for trained professionals. Measures for improvement necessary.

The observation of women’s water lifting and carrying techniques during the transport of water revealed that 28.8% of women kept their backs straight while lifting the container, 60.7% did not bend their knees while lifting, and 37.5% twisted their back while lifting. Table 2 presents further details on water access and water-carrying practices.

### Carrying of other loads

Almost all women were involved in carrying other agricultural loads (92.4%). They started to carry heavy loads including water at a mean age of 11.6 years (SD = 3.7 years). The reported average weight of agricultural loads carried was 35.8kg (SD = 20.6 kg) and exceeded the mean maximum weight of water carried of 24.0 kg (SD = 10.3 kg).

### Water carrying during pregnancy and after delivery

About one third of the women said that they carried the same amount of water during pregnancy and after delivery, while 15.8% said that they were not involved in water carrying during pregnancy and 21.2% during the first three months after delivery, respectively. Slightly more than half of the interviewees said that they carried less water during pregnancy (56.2%) and after delivery (51.8%) (Table 3).

**Table 3:** Water carrying during pregnancy and after delivery.

	N	%	Mean (SD)
During pregnancy, do you carry the same amount of water?	1001		
Yes		28.0	
I carry less		56.2	
I do not carry water		15.8	
3 months after delivery, do you carry the same amount of water?	1001		
Yes		27.0	
I carry less		51.8	
I do not carry water		21.2	
After delivery, how many months should a woman wait to carry water to avoid negative health consequences?	998		4.5 (2.8)
Who helps with lifting heavy loads such as water during pregnancy and after delivery	921		
Husband		52.3	
Mother-in-law/ Daughter-in-law		30.1	
Father-in-law		9.2	
Daughters		6.8	
Sons		2.3	
Other family members		23.2	
Other men in community		4.7	
Other women in community		5.3	
Nobody		20.6	

### Water carrying related risk factors associated with health

**Fig 2:**
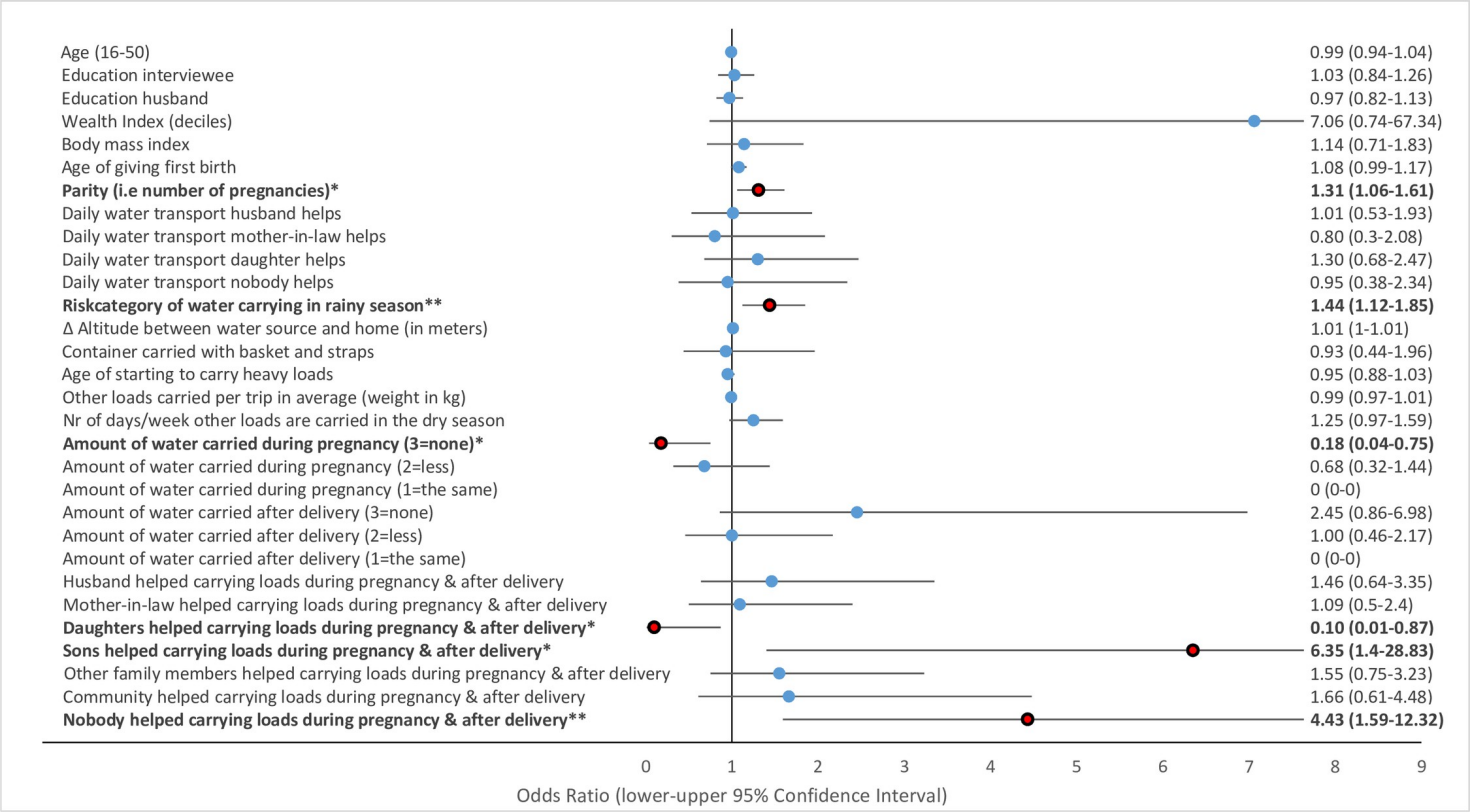
Association of risk factors with strong back pain in multivariate GLMM. (*** = significant at the *p*<0.001-level, ** = significant at the *p* = 0.01-level, * = significant at the *p* = 0.05-level; = odds ratio statistically non-significiant, … = odds ratio statistically significant).

**Fig 3:**
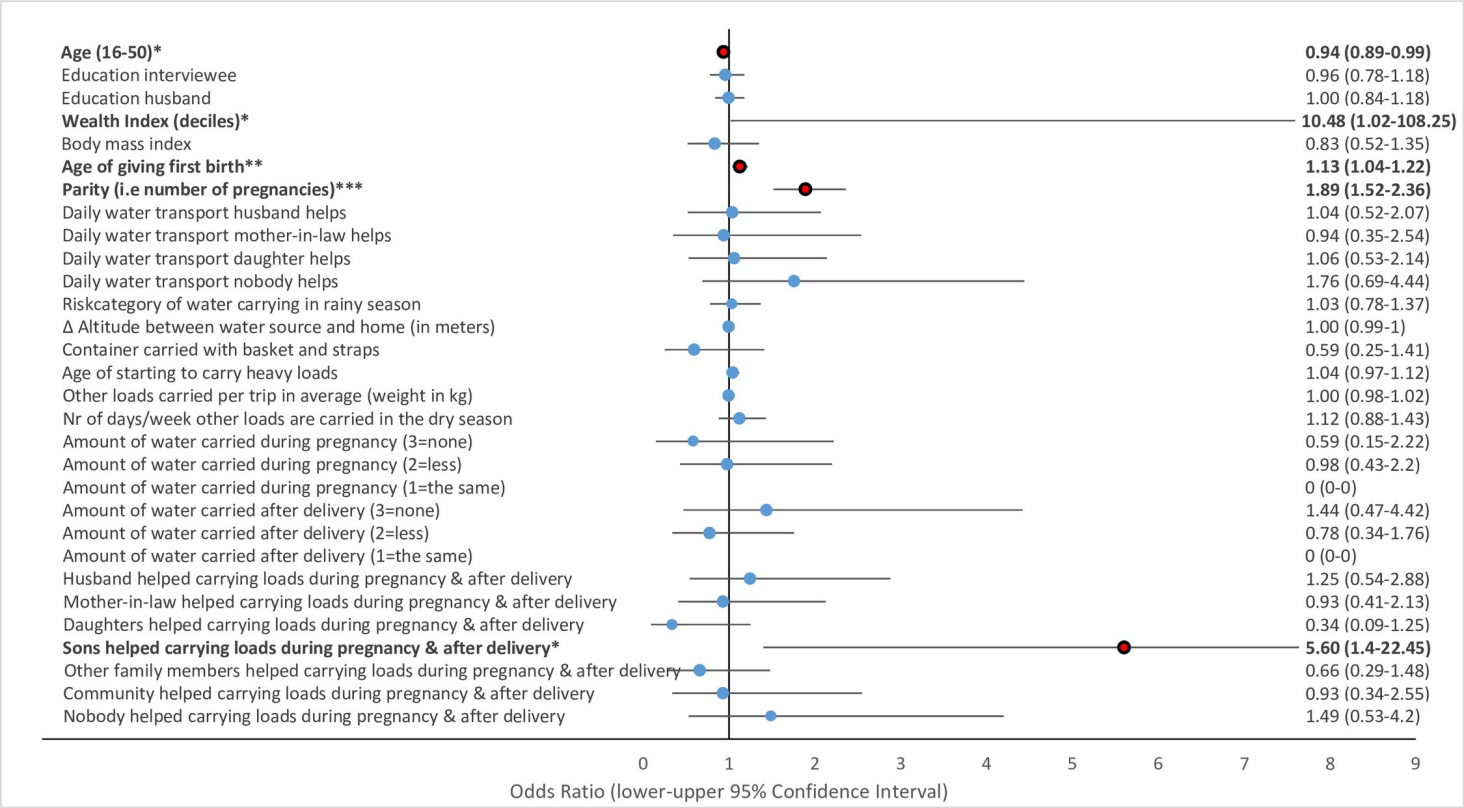
Association of risk factors with pain in the hips in multivariate GLMM. (*** = significant at the *p*<0.001-level, ** = significant at the *p* = 0.01-level, * = significant at the *p* = 0.05-level; = odds ratio statistically non-significiant, … = odds ratio statistically significant).

**Fig 4:**
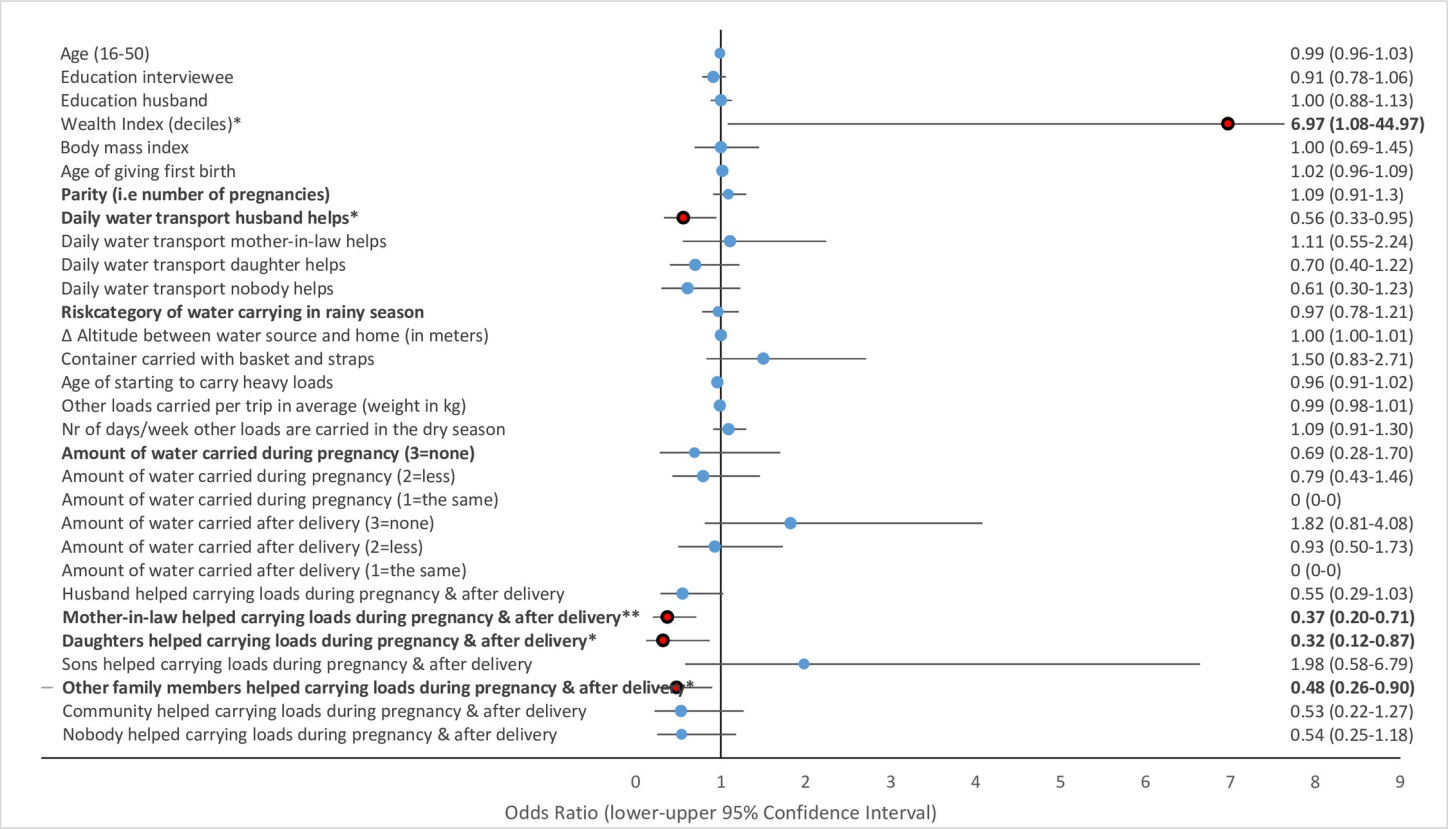
Association of risk factors with pain in the knees in multivariate GLMM. (*** = significant at the *p*<0.001-level, ** = significant at the *p* = 0.01-level, * = significant at the *p* = 0.05-level; = odds ratio statistically non-significiant, … = odds ratio statistically significant).

**Fig 5:**
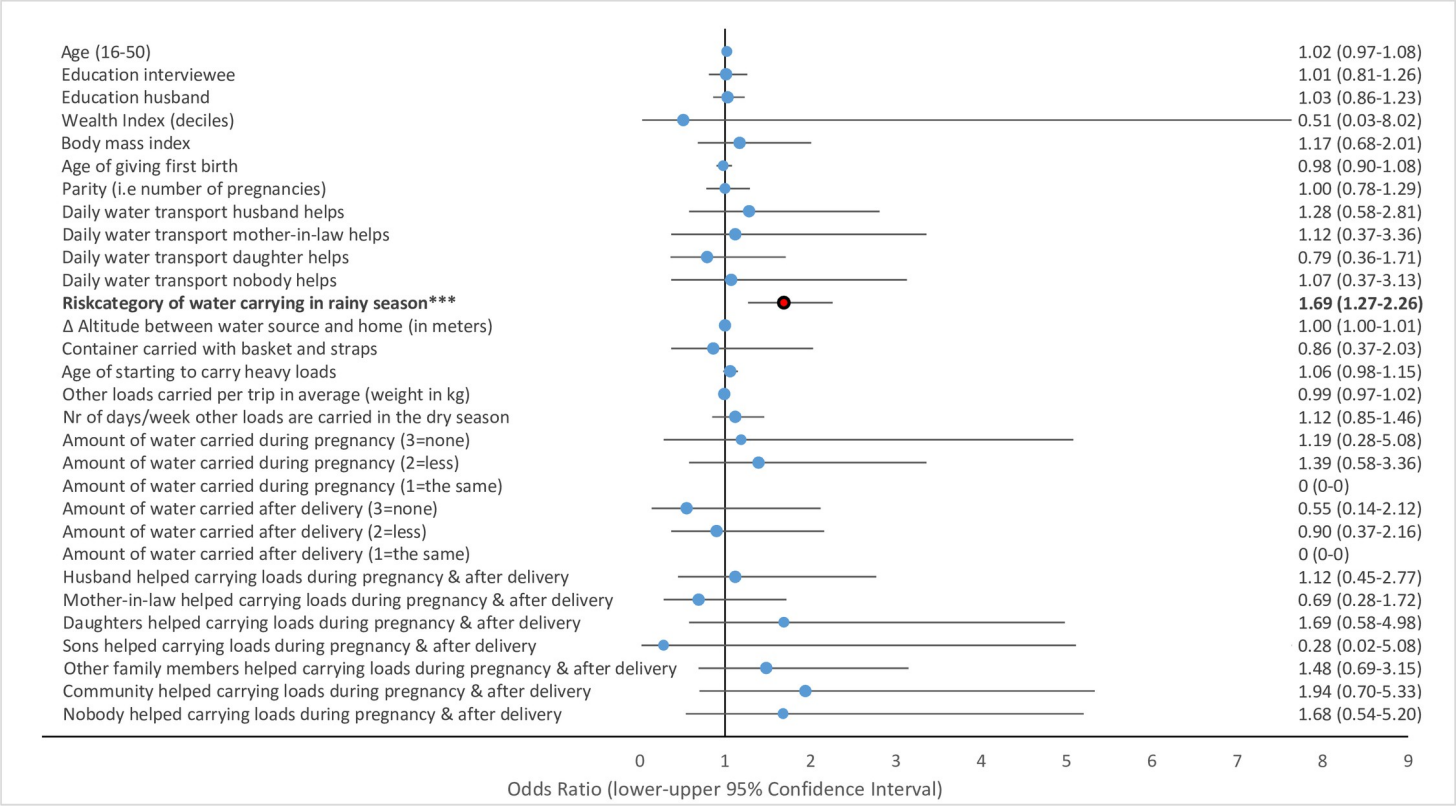
Association of risk factors with uterine prolapse in multivariate GLMM. (*** = significant at the *p*<0.001-level, ** = significant at the *p* = 0.01-level, * = significant at the *p* = 0.05-level; = odds ratio statistically non-significiant, … = odds ratio statistically significant).

**Fig 6:**
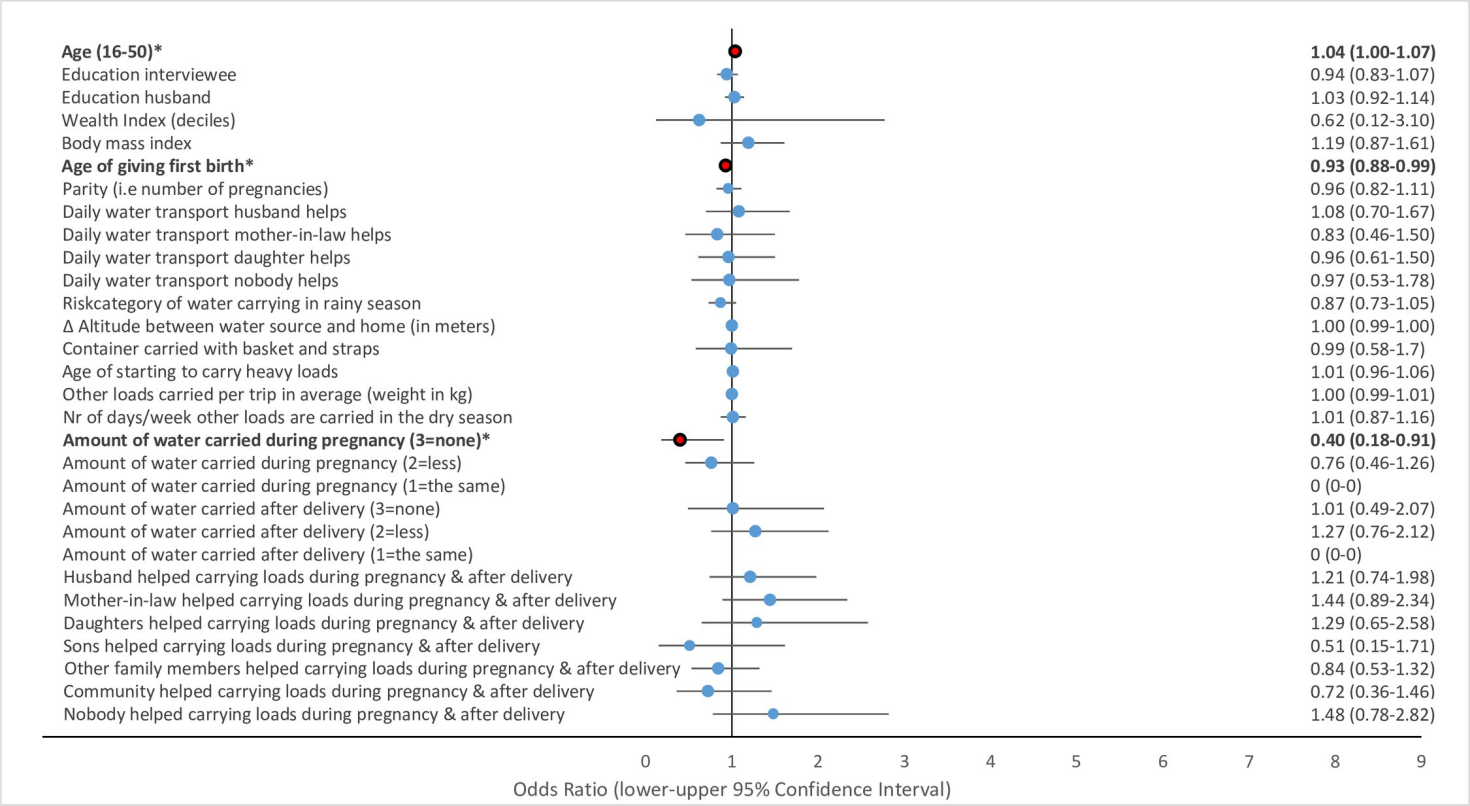
Association of risk factors with spontaneous abortions in multivariate GLMM. (*** = significant at the *p*<0.001-level, ** = significant at the *p* = 0.01-level, * = significant at the *p* = 0.05-level; = odds ratio statistically non-significiant, … = odds ratio statistically significant).

Multivariate GLMM revealed that several water-carrying-related risk factors were associated with physical health problems of women in the study area. Musculoskeletal pains including strong back pain (Fig 2), pain in the hips (Fig 3) and pain in the knees (Fig 4) and uterine prolapse (Fig 5), were significantly associated with various water-carrying-related predictors. Spontaneous abortions (Fig 6) were not significantly associated with water carrying-related predictors. Pain in the neck was not significantly associated with any of the factors included in the model. Data tables of all six models are provided in S4 Table Association of risk factors with uterine prolapse, abortions, strong back pain, neck pain, pain in the hips, pain in the knees in multivariate GLMM in the supplementary materials.

#### Risk factors associated with uterine prolapse

Increasing age and parity are well known risk factors for uterine prolapse [30,48]. Our study confirmed parity as a risk factor and found that increasing parity was associated with higher odds for uterine prolapse at a ratio of 1.31 (OR = 1.31, *p* = 0.011, 95% CI = 1.06–1.61). Age was not identified as a risk factor for uterine prolapse in our study. This could be due to our sample being restricted to women in the reproductive age who have a lower risk for uterine prolapse compared to older women in menopause or post menopause [49].

The risk category of water carrying in the rainy season was associated with a 44% higher odds ratio for uterine prolapse (OR = 1.44, *p* = 0.004, 95% CI = 1.12–1.85). Women stating that they did not carry any water during pregnancy had an 82% lower odds ratio for uterine prolapse (OR = 0.18, *p* = 0.019, 95% CI = 0.04–0.75). Help received for water transport during pregnancy and after delivery was identified as a highly important protective factor. Women not receiving any help during this period had a 4.4 times higher odds ratio (OR = 4.43, *p* = 0.004, 95% CI = 1.59–12.32) for uterine prolapse, whereas support from daughters was associated with a 90% lower odds ratio for uterine prolapse (OR = 0.10, *p* = 0.037, 95% CI = 0.01–0.87). Help received from sons during pregnancy and during the first three months after delivery was associated with an 6.3 times higher odds ratio for uterine prolapse (OR = 6.35, *p* = 0.017, 95% CI = 1.4–28.83). This may indicate that sons provide this support to women who have already suffered uterine prolapse.

#### Risk factors associated with spontaneous abortions

The risk category of water carrying was not significantly associated with spontaneous abortions. A lower economic status was significantly associated with a ten times higher odds ratio for spontaneous abortions (OR = 10.48, *p* = 0.049, 95% CI = 1.02–108.25). Also a higher parity (OR = 1.89, *p*<0.001, 95% CI = 1.52–2.36)and a higher age of giving the first birth were significantly associated with spontaneous abortions (OR = 1.12, *p* = 0.005, 95% CI = 1.04–1.22). In addition, help provided by sons to carry loads during pregnancy and the first three months after delivery was inversely associated with spontaneous abortion (OR = 5.6, *p* = 0.015, 95% CI = 1.4–22.45), indicating that sons were likely to provide help if their mother previously had suffered a spontaneous abortion.

#### Risk factors associated with strong back pain

The risk category of water carrying was not significantly associated with strong back pain. However, help received with daily water carrying from husbands was associated with a 44% reduced odds ratio for strong back pain (OR = 0.56, *p* = 0.031, 95% CI = 0.33–0.95). Similarly, help received with water carrying during pregnancy and during the first three months after delivery from mothers-in-law, daughters, and other family members was associated with a 52–68% lower odds ratio for strong back pain (mothers-in-law: OR = 0.37, *p* = 0.003, 95% CI = 0.20–0.71; daughters: OR = 0.32, *p* = 0.026, 95% CI = 0.12–0.87; other family members: OR = 0.48, *p* = 0.021, 95% CI = 0.26–0.90).

#### Risk factors associated with pain in the hips

The risk category of water carrying in the rainy season was associated with a 1.69 times higher odds ratio for pain in the hips (OR = 1.69, p<0.001, 95% CI = 1.27–2.26). The risk category of water carrying in the rainy season was the only factor included in the model with a significant association with pain in the hips.

#### Risk factors associated with pain in the knees

Our analysis revealed that pain in the knees was significantly associated with the increasing age of the interviewee and a lower age when giving the first birth. The odds ratios of both of these factors though were close to one, indicating a small effect. In addition, women stating that they did not carry any water during pregnancy had a 60% lower odds ratio for pain in the knees (OR = 0.40, *p* = 0.029, 95% CI = 0.18–0.91).

## Discussion

Despite water supply improvements over the past 20 years, our results suggest that carrying water in rural Nepal still is difficult, particularly in the dry season, when 39% of the women in our sample had to use a more distant water source than during the rainy season.

Although previous studies have shown that the involvement of men in carrying water increases with an increasing number of household connections [7], the main responsibility for carrying water to the home in our study area still rested on women. The average weights of water, as well as of other loads carried in the study area were significantly above SUVA’s recommendation that women regularly lift maxima of 10 to 15kg to avoid musculoskeletal disorders [41]. The weights were also above the 22kg threshold recommended by the ILO for short-term lifting [42]. Accordingly, a quarter of women were in the two highest health risk categories defined by SUVA for lifting and carrying loads [43] during the rainy season, and this proportion rose to half during the dry season. In addition to water carrying, our study also assessed the association of the weight of other loads carried with the evaluated health outcomes. Interestingly, we did not find a statistically significant association between the weight of other loads carried and any of the health outcomes, despite of the fact that other loads being carried had higher average weights than the weight of water carried. A potential explanation for this could be that the need to carry other loads was less pressing than the need to secure water for the family and, therefore, women had more flexibility to avoid carrying other loads during periods of high vulnerability and thereby could reduce associated health risks.

Findings on the relation between water carrying and women’s health have previously been summarized by Geere at al. in a systematic review of 42 studies [9]. Our findings provide additional evidence specific to the Nepalese context on the association of water-carrying-related risk factors with musculoskeletal disorders, uterine prolapse and spontaneous abortions.

Musculoskeletal disorders cause an enormous global disease burden with the highest disability and the fourth highest burden of all non-communicable diseases in developing countries. Nevertheless, there are still major gaps in the understanding of prevalence and predictors [50]. Our study found a very high overall prevalence of back pain, 61%, with about 18% of it being horrible to excruciating. The water-carrying-related risk category in our study however was not significantly associated with strong back pain. This was surprising at first sight, but our data does not capture the risk category of water carrying done by women in the past and it may be that women who now complained about strong back pain, are not able to carry heavy loads anymore and therefore, their current water carrying practice is categorized as a lower risk category [51]. Their previous water carrying, however, might have been in a high risk category. An indirect association between water carrying and strong back pain was identified since women receiving support from their husbands in the daily transport of water had a 44% reduced odds ratio for strong back pain. In addition, women receiving support for water carrying during pregnancy and after delivery had a 52–68% reduced odds ratio for strong back pain. A significant association was found between the water carrying-related risk category and pain in the hips, while the association with pain in the neck and pain in the knees was not significant.

The global burden of disease for pain in the neck is also high, accounting for the third highest disability and the eighth highest burden of all non-communicable diseases in developing countries [50]. However, the overall prevalence of pain in the neck was quite low in our study area at 2.9% and none of the water-carrying-related risk factors was significantly associated with pain in the neck. Previous studies among children and adults transporting water on the head in several African countries, found much higher prevalences of pain in the neck: in South Africa 41% of adults and children reported neck pain [52], in Malawi, 25% of children complained about pain in the back and neck [53] and in Nigeria 98.5% of girls indicated neck pain [54]. We think that this difference might be related to different water-carrying practices in Africa, where water containers are frequently carried on the head, while the majority of Nepalese women carry water containers on the waist. Around 10% of women in the study area used a basket and straps tied around the head to carry water containers.

The global prevalence of knee osteoarthritis is estimated at 16.0% for individuals above 15 years and 22.9% above 40 years with substantial differences between countries [55]. Established risk factors for osteoarthritis of the knee are increasing age, obesity, gender, and genetics, and there is increasing evidence that regular lifting of heavy loads is a risk factor [56,57]. Women in our study area had a disproportionately high prevalence of pain in the knees (33.7%). Nevertheless, our study did not identify a significant association with water carrying related risk factors, except that women not carrying any water during pregnancy had a 60% lower odds ratio for pain in the knees.

Symptoms of uterine prolapse were reported by 11.2% of the women interviewed. These symptomatic prevalence values were slightly higher than those of 10% found during reproductive health camps in Nepal [20,21] although our sample only included women in the reproductive age. The prevalence was also higher than in the USA or Europe [23]. Another cross-sectional study in eight districts in Nepal that used questionnaires and clinical examinations found an average prevalence for uterine prolapse of 10% with much higher prevalence values in the flat Terai regions than in the hilly districts [22]. A higher risk category of water carrying in the rainy season was associated with a 44% higher odds ratio for uterine prolapse.

In our project site, 12.8% of the women had experienced at least one spontaneous abortion, this is slightly higher than the national average of about 10.8% across all women in the reproductive age in Nepal [6]. In view of the difficulties of detecting early pregnancies in the first trimester in a remote rural context, we think that actual prevalence at the study sites might be higher than presented; other studies found a 1–2% prevalence of miscarriage after 12 weeks of pregnancy and a general prevalence of 12–15% [58,59]. A large cross-sectional study among 500,000 women in China found a comparable incidence of spontaneous abortion of 9.04% in rural areas and 3.75% in urban areas [60]. Our study did not find an association between BMI and spontaneous abortion. This is in contradiction to previous studies that found a significant association between miscarriage and obesity [61,62] including a study that used data from a national health survey in Nepal [63]. Although about 40 percent of the women in our study site were obese, they were living in a rural and hilly area, which might have obliged them to walk more than women living in urban areas. Consequently, they could have been in a better physical condition, which could have reduced their risk for spontaneous abortions. In our study, none of the water-carrying-related risk factors was significantly associated with spontaneous abortions.

Sorensen at al. noted the need to investigate the association between the terrain and water carrying [64]. Our study was conducted in the difficult terrain of the Nepalese hills where almost half of the women had to carry containers filled with water uphill or downhill. Nevertheless, the topography was not identified as a significant risk factor for water carrying related physical health outcomes among women in the reproductive age, although it was significantly related to higher emotional distress [10].

Women face a high pressure to carry water even if they do not feel well because water is a basic necessity required each day for consumption, hygiene, and providing for the cattle. This dependence upon water increases women’s susceptibility to water-carrying-related health problems, especially during vulnerable periods and could explain why water-carrying-related risk factors were significantly associated with several health outcomes, whereas carrying other agricultural loads was not despite the heavy weights of agricultural loads that were carried.

### Water carrying behavior during pregnancy and during the first three months after delivery

Women in the project area were heavily engaged in water carrying during pregnancy and during the first three months after delivery at the time of our study. The weights carried during these periods far exceeded recommendations for maximum loads of up to 11kg after week 24 during pregnancy [64,65]. The intensive involvement of Nepalese women in heavy agricultural tasks during pregnancy and shortly after delivery has been discussed [13,19]. However, we are not aware of any prior study that has systematically assessed the association of women’s health and water carrying during pregnancy and after delivery and the impact of social support for carrying water during this period.

An important finding of our study is that lacking support for carrying water and other loads during pregnancy and during the first three months after delivery is associated with uterine prolapse and strong back pain among women of reproductive age in the study area. In addition, women who did not carry any water during pregnancy had an 80% reduced odds ratio for uterine prolapse and a 60% reduced odds ratio for pain in the knees.

Our study findings indicated a pressing need to raise awareness on the need to support women who carry water and other loads during pregnancy and after delivery to reduce associated health risks. Particular addressees of such behavior change campaigns should be older women in the household, husbands, and sons so that social support for women would increase during this vulnerable period.

It is expected that the water supply situation in Nepal will further improve in the coming years; the targets of the Government of Nepal under the Sustainable Development Goal 6 for 2030 are to provide basic water supply to 99% of the households and safely managed drinking to 90% [66]. Progress in improving the physical conditions of water supply is required to further reduce the water-carrying-related health risks to women in Nepal.

### Limitations

Most data collected for our study are based on self-reported accounts of health problems and are therefore subject to a possible reporting bias. Estimates of the prevalence and degree of uterine prolapse are based on symptomatic occurrence and are likely to be underestimated due to underreporting because of shame and social stigmatization [67]. Comparison with prevalence in other areas was difficult due to the lack of using standardized definitions for prolapse across studies [27]. Furthermore, the prevalence of spontaneous abortions is likely to be underestimated due to the lack of early diagnosis tools for pregnancy in remote rural settings [59]. Such non-differential misclassification of outcome measurements could have caused a possible information bias leading to a systematic error, underestimating the odds ratios presented in the logistic regression models.

The study used a cross-sectional design; therefore, we can only provide information about the direction and degree of association between risk factors and outcome variables, and cannot make any statements on causality or infer changes over time.

## Conclusions

Despite improvements in the water supply conditions over the past 20 years, we found a high prevalence of strong back pain, pain in the knees, uterine prolapse, and spontaneous abortions among women in the reproductive age in the study area. Uterine prolapse, strong back pain and pain in the hips were significantly associated with several water-carrying-related risk factors, such as support received with daily water carrying, and the risk categories of water carrying, which were defined by the weight carried, the distance, frequency of carrying, and body posture during lifting.

In addition, water carrying during pregnancy and during the first three months after delivery was widely practiced in the study area and indicated that women have little flexibility to avoid carrying water if they do not feel well because water is a daily required basic necessity. Support received for water carrying during pregnancy and after delivery and not carrying any water during pregnancy was associated with reduced odds ratios for uterine prolapse, strong back pain and pain in the knees.

Further improvement of the water supply infrastructure is required to reduce women’s water-carrying-related health burden. In addition, we suggest assessing the effectiveness of behavior change interventions that target social support systems to increase the awareness of the importance of liberating pregnant women from their involvement in lifting loads, such as the daily carrying of water, and increasing social support from them to reduce water-carrying-related health risks [51]. In addition, we propose to more broadly disseminate guidance on the correct posture for lifting loads and on permissible weight limits.

## Supporting information

S1 Questionnaire Observation and health examination form. (XLSX)

S1 Table Measurements of variables. (XLSX)

S2 Table Demographic information. (XLSX)

S3 Table Association of risk factors with uterine prolapse, abortions, strong back pain, neck pain, pain in the hips, pain in the knees in multivariate GLMM. (XLSX)
